# Seasonal shifts in pronghorn antelope (*Antilocapra americana*) diets under a new lens: Examining diet composition using a molecular technique

**DOI:** 10.1371/journal.pone.0292725

**Published:** 2023-10-11

**Authors:** Cole A. Bleke, Eric M. Gese, Shane B. Roberts, Juan J. Villalba

**Affiliations:** 1 Department of Wildland Resources, Utah State University, Logan, Utah, United States of America; 2 U.S. Department of Agriculture, Wildlife Services, National Wildlife Research Center, Utah Field Station, Logan, Utah, United States of America; 3 Idaho Department of Fish and Game, Boise, Idaho, United States of America; Universitat Autonoma de Barcelona, SPAIN

## Abstract

Foraging is one of the most fundamental activities contributing to the maximization of an animal’s fitness, and thus herbivores must optimize their diet selection and intake to meet their nutrient demands for survival, growth, and reproduction. Using plant DNA barcoding, we determined diet composition of five subpopulations of adult female pronghorn antelope (*Antilocapra americana*) grazing rangelands in southern and southeastern Idaho, USA. Fecal samples were collected for two years (2018–2019), and across metabolically-important adult female life history stages (late gestation, early lactation, breeding season). Plant DNA barcoding yielded 137 detected species within pronghorn diets across subpopulations and sampling periods with forbs being the most abundant. Pronghorn dietary functional group composition ranged from 52.2–60.3% from forbs followed by shrubs (22.6–28.2%), graminoids (8.7–15.7%), and legumes (5.5–9.6%). Dietary protein intake was also highest from forbs and ranged from 32.4–62.4% followed by graminoids (1.2–43.1%), shrubs (18.7–21.3%), and legumes (2.6–7.4%). We found significant intra- and interannual differences in the mean number of genera-based plant detections in pronghorn diets. Dietary protein intake of cultivated legumes (e.g., alfalfa [*Medicago sativa*] and sainfoin [*Onobrychis viciifolia*]) was lower than expected, ranging from <1.0–30.8%, suggesting that even within an agricultural-dominated landscape, factors other than plant nutritional composition contributed to pronghorn diets. Although the plant DNA barcoding technique exhibits limitations, it demonstrated potential for elucidating pronghorn dietary species richness, particularly for plants consumed in small proportions, as well as for observing temporal fluctuations in functional group composition and dietary protein intake explained through the interplay between environmental factors, plant chemical composition, and the animals’ physiological needs.

## Introduction

The consumption of food is one of the most fundamental activities because animals must optimize diet and intake to meet their nutrient demands for survival, growth, and reproduction [1]. Nutritional status influences maternal body condition, pregnancy, body size, and survival [2], and this key variable is impacted by changes in forage species availability, abundance, and plant phenological stage [3]. Therefore, assessing the composition of wild and domestic ungulate diets has long been of interest to range and wildlife ecologists [4]. Understanding food choice is also key to investigations of the potential impacts of ungulates on ecosystems and human resources (e.g., agricultural lands), since satisfying dietary needs is one of the strongest drivers of ungulate behavior [5]. Ungulates experience energy-demanding cycles of reproduction coupled with periods of nutritional restrictions and climatic stress [6] that warrant detailed studies of dietary composition both spatially and temporally.

Pronghorn antelope (*Antilocapra americana*) are often described as intermediate or mixed feeders that are selective and opportunistic with regards to the forage they consume. These animals have a small rumen that limits their ability to digest plants with high amounts of lignocellulose, resulting in avoidance of plants rich in this component such as graminoids, and preference for forages with greater amounts of cell contents such as forbs and shrubs [7]. Their diets can be diverse and vary with the palatability, availability, succulence, and nutritional gains present in the plant species available in their environment, notably forbs, grasses, and shrubs [8]. Diverse diets are important because there are positive associative effects from protein-rich forages (e.g., forbs) in that allow for more efficient utilization of plants high in fiber, (e.g., graminoids; [9]).

Traditional methods for herbivore diet determination have included direct and indirect observations, assessments of feeding traces along transects, and rumen and fecal analyses [8]. While over 200 pronghorn diet studies have been completed, microhistological analysis of fecal matter has been the primary method used for the past 50 years [8, 10]; where composition of the diet is determined based on identifiable plant fragments in the feces [11]. Microhistology presents potential limitations in accuracy due to vegetation identification constraints, differential digestion of plant species, and human error given it is a labor-intensive process that requires extensive training [12]. Some key considerations when using microhistology is that highly digestible plants (e.g., forbs) are likely to be underestimated [12, 13] and “low frequency” plants are less likely to be discovered and quantified [14, 15].

An alternative and emerging method is the use of plant DNA barcoding [16] which involves amplifying chloroplast trnL from a genomic DNA sample to determine plant species or genera present in the diet. The abundance of plant chloroplast is a representation of the relative protein content of that plant species or genera [12]. Sequenced DNA is matched against a reference database to identify plant taxa [10, 17], which may only require small amounts of plant matter to sequence and, therefore, potentially increasing its accuracy of plant species identification in a diet [12].

The overall objective of our study was to explore the use of plant DNA barcoding at showcasing pronghorn diets from noninvasively-collected fecal samples. We quantified mature female pronghorn antelope seasonal dietary plant functional group composition and dietary protein intake from each functional group. We hypothesized that pronghorn (1) would display a greater temporal consumption of forbs beyond peak growing season, (2) would seasonally shift their dietary protein intake to accommodate differing metabolic demands and changes in plant phenology, and (3) would consume differing proportions of plants across sampling periods given their flexibility and adaptability as intermediate feeders.

## Materials and methods

### Study area

**Fig 1:**
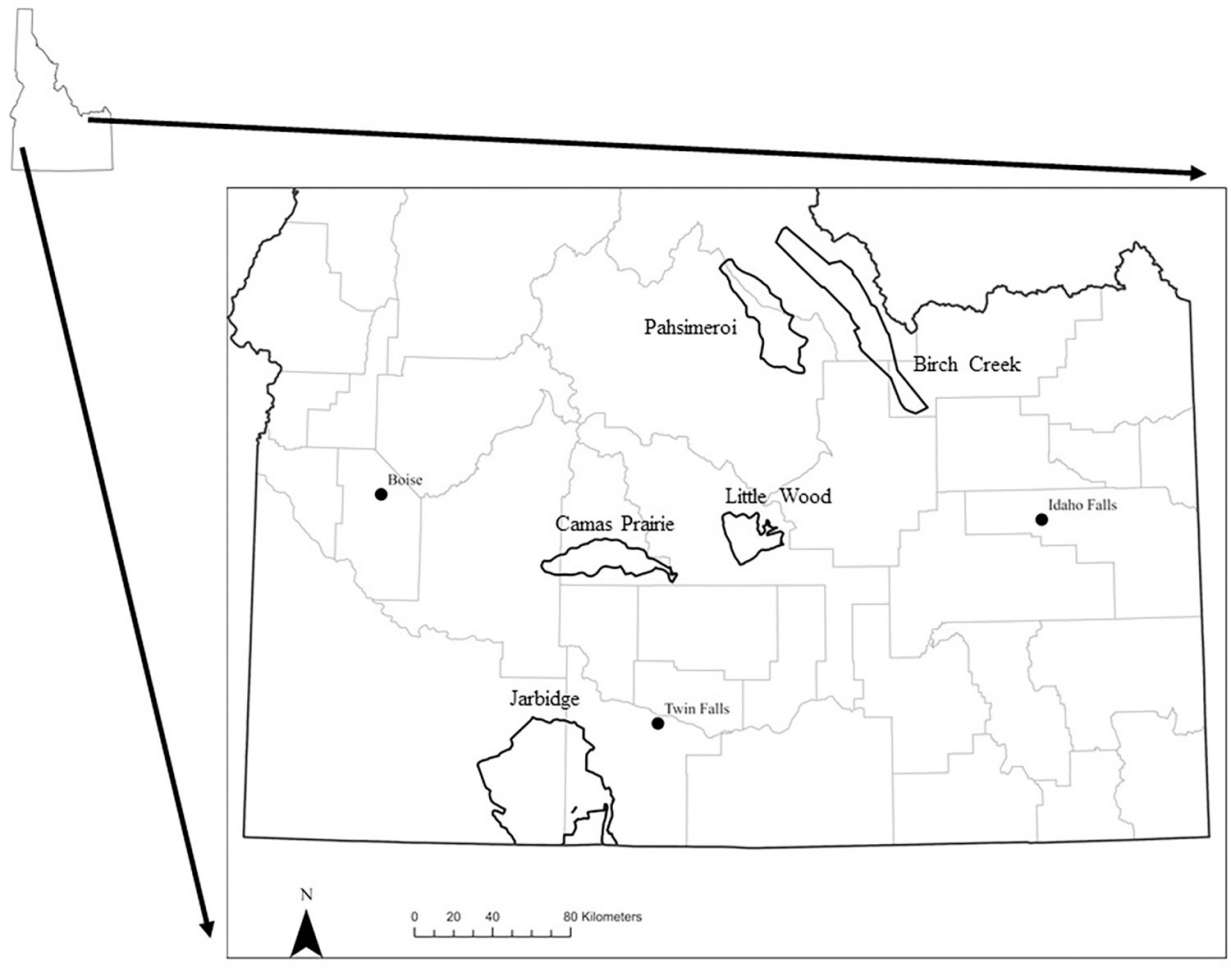
Pronghorn antelope summer distributions. Ranges of the five study subpopulations (Birch Creek, Camas Prairie, Jarbidge, Little Wood, Pahsimeroi) of pronghorn antelope within the state of Idaho. Grey lines represent county boundaries.

The subpopulations we studied represented the majority of pronghorn habitats and population productivities in southern Idaho, USA (Fig 1; [18]). Study subpopulations included Jarbidge, Camas Prairie, Little Wood, Birch Creek, and Pahsimeroi. The Jarbidge study site represented a resident pronghorn subpopulation occupying desert habitat. Basin and Wyoming sagebrush (*Artemisia tridentata tridentata* and *A*. *t*. *wyomingensis*) were the dominant cover types, which encompassed >60% of the study area [19]. Perennial grasslands were the next most dominant cover type, comprising 25% of the area. The remainder of the site was a mix of low sagebrush (*Artemesia arbuscula*), antelope bitterbrush (*Purshia tridentata*), and rabbit brush (*Chrysothamnus* spp.) communities [18]. There was minimal irrigated agriculture within this site. The Jarbidge area received an average annual precipitation was 37.64 cm with average annual maximum and minimum temperatures of 15.46°C and 1.40°C [20], respectively, and mean elevation is 1,552 m.

The Camas Prairie study site typified a migratory pronghorn persisting largely on converted agricultural lands through the summer months. The majority of the study area was under agricultural cultivation (~50%), of which 81% was dryland and 19% was irrigated. Alfalfa (*Medicago sativa*) was the dominant crop (54% of agricultural area), followed by barley (*Hordeum vulgare*; 13%) and grass hay (11%; [21]). The remaining agricultural lands were pasture or enrolled in the Conservation Reserve Program. Perennial grasslands (21% of study area) and basin and Wyoming big sagebrush communities (18%) persisted on Bureau of Land Management and state-held lands [18]. The Camas Prairie area received an average annual precipitation of 41.64 cm with average annual maximum and minimum temperatures of 14.0°C and -0.23°C [20], respectively, and mean elevation is 1,552 m.

The Little Wood study site was selected to characterize migratory pronghorn primarily occupying native shrub steppe range. Local ranches grazed domestic cattle (*Bos taurus*), sheep (*Ovis aries*), and horses (*Equus ferus caballus*) throughout the area. Basin and Wyoming big sagebrush covered 73% of the study area. Agricultural land accounted for 6% of the study area, with irrigated alfalfa being the primary crop. The remainder of the study area consisted of mountain big sagebrush (*A*. *t*. *vaseyana*), perennial grasslands, and antelope bitterbrush cover types [18]. The Little Wood area received an average annual precipitation of 40.02 cm and experienced an average annual maximum and minimum temperature of 13.56°C and -1.19°C [20], respectively, and mean elevation is 1,726 m.

The final two study sites, Birch Creek and Pahsimeroi, represented migratory pronghorn inhabiting mountain valley habitats [18]. These subpopulations likely overwintered together but were separated during the summer by the Lemhi mountain range [22]. The Birch Creek subpopulation occupied the Birch Creek and Lemhi valleys, where low sagebrush was the dominant vegetation community encompassing 51% of the area. Mountain, basin, and Wyoming big sagebrush accounted for another 40% of the study site, with limited agricultural lands in the valleys (4%) and interspersed forest stands (4%; [18]). The Birch Creek area received an average annual precipitation of 39.80 cm with an annual maximum temperature of 11.36°C and annual minimum temperature of -2.74°C [20] and mean elevation of 2,018 m. The Pahsimeroi subpopulation was located within the Pahsimeroi and Little Lost River valleys, where mixed stands of mountain big sagebrush and low sagebrush dominated much of the landscape (>60%). Basin and Wyoming big sagebrush were the next most abundant cover types and comprised 23% of the study area combined. Agricultural cropland accounted for <2% of the study area [18]. The Pahsimeroi area received an average annual precipitation of 33.02 cm with an annual maximum temperature of 12.45°C and annual minimum temperature of -2.39°C [20] and mean elevation of 1,897 m.

### Sampling method

We collected fresh fecal samples from unmarked, reproductive-aged, female pronghorn (i.e., ≥2 years) in 2018 and 2019 during three sampling periods selected to coincide with metabolically-demanding maternal life history stages: late gestation (April to mid-May), early lactation (June), and breeding season (September). We were unable to associate pregnancy or lactation status of adult female pronghorn with samples, given our sampling design; therefore, we discuss our results in accordance with female life history stages. We used magnifying optics to categorize individuals by age class and sex and to monitor defecation. Once defecation occurred, we used 2-person teams with two-way radio communication to locate pellet piles. If we were uncertain whether an individual female pronghorn was sexually mature (e.g., lone individuals that lacked a reference for age or size or individuals perceived to be yearlings based on shoulder height or muzzle length), we did not collect a sample. We made a concerted effort to collect fresh fecal samples only from spatially-segregated groups of animals to obtain a representative sample of the subpopulation and avoid re-sampling.

### Laboratory methods

We collected a total of 1,440 samples combined across years, sampling periods, and sites. From these specimens, 20 samples/subpopulation/sampling period/year (560 samples total; 260 from 2018, 300 from 2019) were randomly selected. Samples were placed in a drying oven (Precision Scientific, Chicago, IL), where temperatures were held below 50°C, until all moisture was evaporated from the fecal samples. Dried samples were then ground using a coffee grinder (Hamilton Beach, Southern Pines, NC) until fecal material became a consistent powder in texture. Dried and ground samples were sent to Jonah Ventures laboratory (Boulder, CO) for plant DNA barcoding analyses with details of laboratory methodologies summarized by [23].

Briefly, laboratory results for each individual sample and associated Exact Sequence Variants (ESV) were compiled into a table including sequences and read counts for each sample. Sequencing success and read quality were verified using FastQC v0.11.8 and reads were demultiplexed by using Illumina-utils v2.6 (iu-demultiplex) using default settings [23]. Reads affected by sequencing and PCR errors were removed using the unoise3 algorithm with an alpha value of 5 to prevent operational taxonomic unit (OTU) clustering [24]. Taxonomic precision and certainty were assigned to each ESV by mapping them against a GenBank reference database [25], as well as Jonah Ventures’ voucher sequence records, using usearch_global with -maxaccepts 0 and -maxrejects 0 to ensure mapping accuracy. Consensus taxonomy was generated from the hit tables, by first considering 100% matches, and decreasing in intervals of 1% [23]. We removed all ESVs with <95% match from final statistical analyses, resulting in 60.31% of all reads being included. Relative read abundances of results for each sample were standardized by functional group and divided by the total number of detections, provided by the lab, to calculate a percent breakdown of each functional group. The linkage between relative read abundance and relative protein intake is based on concentration of chloroplast DNA detected per ESV [23]. Each ESV was assigned to a representative genus if multiple detections of species of the same genera matched the ESV, and assigned to a plant functional group.

Functional group classifications included: graminoids (monocotyledons such as grasses, sedges, rushes), legumes (species within the Fabaceae family), forbs (non-leguminous herbaceous eudicots), shrubs (non-leguminous woody eudicots), and other (species within Asteraceae, Orobanchaceae, and Rosaceae families). We characterized legumes as a separate functional group from forbs and shrubs because we wanted to quantify dietary protein intake of agricultural crops. From this, we generated OTUs to report, dependent upon the taxonomic specificity of diet results, that represented distinct plant DNA detections. We combined multiple OTUs of the same family or genus for analyses. An OTU represents the relative protein content of the plant, rather than biomass [12]. We calculated the overall percentage component of the diet, for functional group or family- and genus-level OTUs, by summing all samples and dividing by the total. We reported results at the genus level and averaged across samples within a subpopulation, sampling period, and year to calculate the contribution (%) of each OTU to the overall diet. We defined the overall diet as all OTUs that comprised a minimum of 1% of the diet for at least one subpopulation per sampling period and year.

### Data analyses

We used a generalized linear mixed model with a Poisson distribution in R [26] to examine differences in pronghorn diet diversity across time; with subpopulation as the random effect, year and sampling period and their interaction (i.e., period*year) as fixed effects, and the number of genus-level OTUs as the dependent variable. We used a Bonferroni post-hoc test for multiple comparisons since it is a conservative approach and has more power when the number of comparisons is small. We did not test for a difference in the number of graminoid OTUs because only one genus was detected for all sample periods and both years. We used an alpha level of 0.05 for statistical significance.

## Results

For the fecal specimens collected, we removed one sample due to lack of adequate, amplifiable chloroplast DNA and a second sample due to contamination, resulting in 558 useable fecal specimens. Plant DNA barcoding analysis identified 214 OTUs at varying taxonomic levels (32 families, 45 genera, 137 species). The number of OTUs consumed by pronghorn fluctuated by sampling period from 110 to 126 to 115 from late gestation to early lactation to breeding season, respectively. Within that range, forb OTUs were the most abundant in pronghorn diets; with 58, 77, and 61 detected during late gestation, early lactation, and breeding season, respectively. Diversity of shrub genera consumed was highest during late gestation (31 genera), forb diversity was highest during early lactation (77 genera), and grass and legumes diversity was highest during the breeding season (18 and 11 genera, respectively).

We consolidated these results further by selecting for OTUs that comprised at least 1% of any subpopulations’ protein intake in any sampling period which reduced the total number of OTUs to 59 (14 families, 14 genera, 31 species; Table 1). Of the 45 genera-level OTUs, 28 were forbs, 1 graminoid, 7 legumes, 1 moss, and 8 shrubs. The most consistent detections within each functional group were cushion buckwheat (*Eriogonum ovalifolium*) and prostrate knotweed (*Polygonum aviculare*) within forbs; *Bromus* spp. within graminoids; alfalfa and sainfoin within legumes; and sagebrush and antelope bitterbrush within shrubs. We grouped family-level detections into an “other” category for each functional group (i.e., forb, graminoid, legume, shrub), which ranged from 1 to 7 families. Poaceae was the sole family detected within “Other graminoids,” as was Fabaceae within “Other legumes”. Oleaceae and Salicaceae were the two families detected within the “Other shrubs” category. The “Other forbs” category contained Apiaceae, Brassicaceae, Convolvulaceae, Malvaceae, Onagraceae, and Polygonaceae families. These “other” categories for each functional group ranged from < 0.10% to 76.89% of total protein intake recorded. We also created a separate “other” category that contained the families Orobanchaceae, Rosaceae, and Asteraceae due to the fact that each contains forbs and shrubs. This prevented us from classifying them to a specific functional group which resulted in diet breakdowns not summing to 100%.

**Table 1:** Diet composition of pronghorn antelope in Idaho by plant DNA barcoding analysis.

	Birch Creek						Camas Prairie					Jarbidge						Little Wood					Pahsimeroi					
	LG		EL		BS		LG	EL		BS		LG		EL		BS		LG	EL		BS		LG		EL		BS	
	2018	2019	2018	2019	2018	2019	2019	2018	2019	2018	2019	2018	2019	2018	2019	2018	2019	2019	2018	2019	2018	2019	2018	2019	2018	2019	2018	2019
Total forbs:	21.2	27.6	85.5	69.5	47.0	13.2	57.6	70.6	77.8	65.6	39.0	8.3	36.9	73.2	47.6	37.6	30.6	54.7	31.9	46.0	60.6	18.9	38.6	14.2	65.1	56.9	30.6	41.6
*Amsinckia*	-	-	-	-	-	-	0.2	2.3	0.2	0.4	-	-	-	-	-	-	-	-	0.2	-	-	-	-	-	-	-	-	-
*Bassia*	0.1	0.1	0.7	1.6	0.4	-	-	0.6	-	-	-	-	-	0.1	0.1	0.5	-	-	-	-	-	-	-	-	4.4	1.6	0.1	4.6
*Blitum*	-	-	8.0	1.2	0.1	-	-	-	-	-	-	-	-	-	0.7	-	-	-	0.1	0.6	-	-	-	-	0.5	-	-	-
*Boykinia*	1.2	0.2	0.3	0.1	-	-	0.1	-	-	-	-	0.4	0.1	-	-	-	-	0.4	-	-	-	-	1.2	-	0.1	-	-	-
*Castilleja*	-	-	2.5	2.4	3.4	-	-	-	-	-	-	-	-	0.3	0.2	-	-	-	2.7	0.6	-	0.4	-	-	-	1.1	-	-
*Cerastium*	-	-	-	-	-	-	-	-	-	-	-	-	6.1	-	-	-	-	-	-	-	-	-	-	-	-	-	-	-
*Chenopodium*	-	-	0.3	-	-	0.4	0.1	0.2	0.2	0.1	-	-	-	-	0.2	-	-	-	-	0.7	0.5	-	-	-	0.6	2.7	-	0.1
*Chrozophora*	-	-	-	-	-	-	-	-	-	-	-	-	-	5.7	-	-	-	-	0.1	-	-	-	-	-	-	-	-	-
*Chloropyron*	-	0.1	0.1	0.2	6.2	0.2	-	-	-	-	-	-	-	-	-	-	-	-	-	-	-	-	-	-	-	-	-	-
*Comandra*	-	0.1	2.4	11.8	7.6	1.9	-	0.5	-	-	-	-	2.3	0.4	1.3	-	-	-	-	-	-	-	-	-	-	5.6	-	-
*Crepis*	-	-	0.1	0.4	-	-	0.1	0.3	-	-	-	0.1	6.9	0.3	1.9	0.1	-	1.4	-	0.6	-	-	0.3	-	-	0.1	-	-
*Descurainia*	-	-	-	-	-	-	-	-	-	-	-	-	-	5.6	0.1	-	-	-	-	-	-	-	-	-	1.0	-	-	-
*Erigeron*	-	-	0.2	-	-	-	-	-	-	-	-	-	-	-	0.1	-	-	-	-	-	-	-	0.1	0.6	3.5	9.5	-	-
*Eriogonum*	18.5	21.1	57.0	36.3	22.3	9.0	15.9	0.7	0.1	-	-	3.7	13.0	2.1	16.7	0.3	4.8	44.8	18.6	11.6	0.5	13.3	24.9	12.0	9.6	20.4	15.8	22.6
*Erodium*	-	-	-	-	-	-	-	-	-	-	-	0.1	2.7	-	-	-	0.5	0.7	-	-	-	-	0.6	-	-	-	-	0.1
*Fallopia*	-	-	-	1.3	-	0.1	3.0	10.4	12.0	5.5	0.1	0.1	0.5	1.1	-	0.1	-	1.7	0.4	13.0	1.5	2.7	0.2	-	0.8	-	-	0.4
*Geum*	-	4.0	0.2	2.2	0.4	-	-	-	-	-	-	0.3	0.3	-	0.5	-	-	0.4	0.1	-	-	-	1.6	-	-	-	-	-
*Lappula*	-	-	-	-	-	-	-	-	-	-	-	-	-	-	-	-	-	-	-	-	-	-	4.1	-	-	0.1	-	-
*Lapsana*	-	-	-	-	-	-	-	1.1	-	-	-	-	-	-	-	-	-	-	-	-	-	-	-	-	-	-	-	-
*Penstemon*	-	0.3	0.2	0.5	0.7	0.2	0.1	-	-	-	-	-	0.3	0.1	0.2	-	-	0.1	-	0.1	-	0.1	1.3	0.3	0.6	-	-	-
*Phlox*	0.1	0.1	-	0.3	0.4	0.1	0.3	-	-	-	-	1.0	0.8	0.5	0.1	-	0.2	-	-	-	-	-	0.1	0.4	-	0.1	-	0.1
*Plantago*	-	-	-	-	-	-	-	-	-	-	-	-	-	-	-	-	-	-	-	-	0.5	-	-	-	-	-	4.3	0.8
*Polygonum*	0.9	-	1.1	0.9	3.4	0.5	14.6	37.6	51.7	51.8	30.7	1.0	-	26.1	2.0	27.1	6.7	-	2.8	2.0	39.3	0.6	0.4	-	15.7	1.3	4.6	11.7
*Potentilla*	-	-	-	4.4	0.3	0.3	2.1	0.6	1.3	0.1	0.1	-	1.3	4.0	0.2	-	0.1	0.1	1.1	0.3	6.1	0.4	0.1	-	0.4	0.1	-	-
*Sedum*	-	0.6	1.7	0.6	0.5	-	-	-	-	-	-	-	-	-	-	-	-	-	-	-	-	-	-	-	0.3	0.5	-	-
*Silene*	-	0.1	0.2	-	0.3	-	-	-	-	-	-	-	-	0.1	0.4	-	-	-	-	0.2	-	-	0.5	-	6.3	3.6	3.3	0.7
*Thlaspi*	-	-	-	-	-	-	-	-	-	-	-	-	-	-	-	-	-	-	-	-	4.9	-	-	-	-	-	-	-
*Viola*	-	-	0.6	0.4	-	-	0.2	-	-	-	-	-	0.1	-	-	-	-	2.1	0.1	2.9	-	-	-	-	-	-	-	-
Other forbs:	0.2	0.9	8.5	4.4	0.3	0.2	19.9	16.0	11.8	7.1	7.5	0.6	0.9	25.0	21.9	9.0	17.6	1.6	5.4	11.9	6.7	1.2	2.3	0.3	19.9	9.7	1.7	0.1
Total graminoids:	53.4	57.0	0.3	0.4	3.2	8.4	5.2	0.3	0.6	1.1	0.1	79.9	36.8	0.3	2.0	1.8	1.1	26.3	1.4	2.1	6.7	0.5	34.4	51.6	0.9	3.6	8.7	15.2
*Bromus*	-	-	-	-	-	-	-	-	-	-	-	1.8	1.9	-	0.1	-	0.5	-	-	-	-	-	0.1	0.2	-	-	-	-
Other graminoids:	52.6	56.8	0.3	0.4	3.2	8.2	4.8	0.1	0.5	0.2	-	76.9	34.0	0.2	1.9	1.8	0.6	25.6	1.2	1.4	6.1	0.4	34.1	49.7	0.2	3.3	8.5	14.9
Total legumes:	0.1	0.1	0.8	9.5	2.0	34.8	16.7	0.8	1.5	4.1	2.8	0.8	0.9	1.3	1.0	0.1	0.1	3.9	0.7	0.8	12.3	0.6	0.5	0.3	8.0	1.8	11.1	6.2
*Hedysarum*	-	-	-	-	-	3.8	-	-	-	-	-	-	-	-	-	-	-	-	-	-	-	-	-	-	-	-	-	-
*Lathyrus*	-	-	-	-	-	-	0.5	-	-	-	-	-	0.4	-	-	-	-	3.8	-	-	-	-	-	-	-	-	-	-
*Lotus*	-	-	-	-	-	-	-	-	0.2	1.5	1.9	-	-	-	-	-	-	-	-	-	-	-	-	-	-	-	-	-
*Medicago*	-	-	0.1	-	0.6	-	0.7	0.3	0.9	2.5	0.8	-	-	0.4	0.6	0.1	0.1	-	-	-	5.8	-	0.4	0.2	5.4	1.0	6.9	5.4
*Onobrychis*	-	0.1	-	9.0	-	30.8	15.4	-	-	-	-	0.2	0.1	0.7	-	-	-	-	-	0.6	-	0.5	0.1	-	-	-	1.7	0.2
*Pisum*	-	-	-	-	-	-	-	-	-	-	-	-	-	-	-	-	-	-	-	-	-	-	-	-	2.4	-	-	-
*Trifolium*	-	-	-	0.2	0.7	-	-	0.2	0.4	-	-	-	-	-	-	-	-	-	0.7	0.2	6.2	0.1	-	-	-	-	2.4	0.4
Other legumes:	-	-	-	-	-	0.1	-	-	-	-	-	-	-	-	-	-	-	-	-	-	0.2	-	-	-	0.1	-	0.1	0.2
Total shrubs:	24.7	14.9	8.2	10.0	25.2	27.8	15.8	13.9	2.5	8.3	0.6	9.4	19.5	5.9	24.6	6.2	13.9	7.5	54.7	33.9	2.7	51.0	24.1	33.3	22.7	23.2	43.0	33.8
*Artemisia*	22.4	12.2	7.0	5.6	22.6	26.4	2.4	0.5	0.1	0.3	0.1	6.9	13.4	2.3	2.7	4.1	13.1	1.3	2.0	2.2	2.4	0.7	22.0	29.4	22.1	19.4	42.1	31.2
*Atriplex*	0.8	2.7	0.2	0.1	0.8	1.0	-	-	0.1	0.1	-	0.8	0.7	0.1	-	0.1	0.6	-	-	-	-	-	0.4	3.2	-	0.3	0.4	0.3
*Cercidiphyllum*	-	-	-	-	-	-	0.1	-	-	-	-	1.2	2.7	-	-	-	-	5.6	-	-	-	-	-	-	-	-	-	-
*Juniperus*	-	-	-	-	-	-	0.1	-	-	-	-	-	2.3	-	-	-	-	0.2	-	-	-	-	-	-	0.1	-	-	-
*Pinus*	1.0	-	0.2	3.0	0.1	-	0.2	0.1	0.9	-	-	-	-	0.1	1.3	-	-	0.1	0.5	2.8	0.1	-	-	-	0.2	1.8	-	-
*Purshia*	-	-	-	-	-	0.1	0.7	0.5	-	-	0.5	-	0.3	2.4	20.5	1.9	-	0.2	51.5	28.4	-	49.0	-	-	-	-	0.1	2.0
*Ribes*	-	-	-	-	-	-	7.4	0.3	0.1	-	-	-	-	0.1	-	0.1	-	0.2	0.4	0.1	0.1	0.1	1.2	0.2	-	0.3	-	0.1
*Salix*	-	-	-	-	0.7	-	0.6	0.6	0.2	1.8	-	-	-	-	-	-	-	-	0.1	0.2	-	-	-	-	-	0.1	-	-
Other shrubs:	0.1	-	-	0.4	-	-	4.0	12.0	0.9	6.0	-	-	-	0.1	-	-	-	-	-	-	-	1.0	-	-	-	-	0.3	-
Other:	0.6	0.3	5.2	10.7	22.2	15.7	4.7	14.4	17.3	20.9	57.5	1.0	5.5	19.3	24.8	54.1	53.2	7.6	11.2	17.0	17.8	29.0	2.4	0.6	3.2	14.5	6.4	3.1
Asteraceae	0.4	0.1	3.3	6.3	4.3	8.4	4.0	14.2	14.6	20.8	57.5	1.0	5.5	18.4	23.4	53.8	53.1	6.9	10.4	10.8	16.9	8.4	2.4	0.6	3.0	2.1	5.8	1.7
Orobanchaceae	0.2	0.2	1.5	1.7	17.5	0.8	-	-	-	-	-	-	-	0.6	0.1	-	-	-	0.3	-	-	0.1	-	-	-	2.3	-	-
Rosaceae	-	-	0.4	2.7	0.4	6.5	0.7	0.2	2.6	-	-	-	-	0.3	1.2	0.2	-	0.6	0.6	6.1	0.8	20.6	-	-	0.2	10.1	0.6	1.5

Relative proportions of protein intake of pronghorn antelope fecal samples from five subpopulations across three sampling periods (Late gestation [LG], Early lactation [EL], Breeding season [BS]), Idaho, USA. Percentage composition of each plant genus and ‘Other’ were summed across all samples per year and sampling period. Total percentage of each functional group was formed from the sum across all samples as a percentage of the total. Results are sorted in alphabetical order. Plant names are given as reported by the laboratory.

The largest coefficient of variation (CV) of forb dietary protein intake across subpopulations and years occurred during late gestation sampling at 54.64% in 2018 and 42.85% in 2019. Early lactation sampling contained the highest CV of legumes dietary protein intake at 137.34% in 2018 and 126.64% in 2019. Finally, dietary protein intake of grasses and shrubs had the largest CVs during breeding season sampling at 76.17% and 130.75% for grasses and 98.75% and 75.72% for shrubs in 2018 and 2019, respectively.

**Fig 2:**
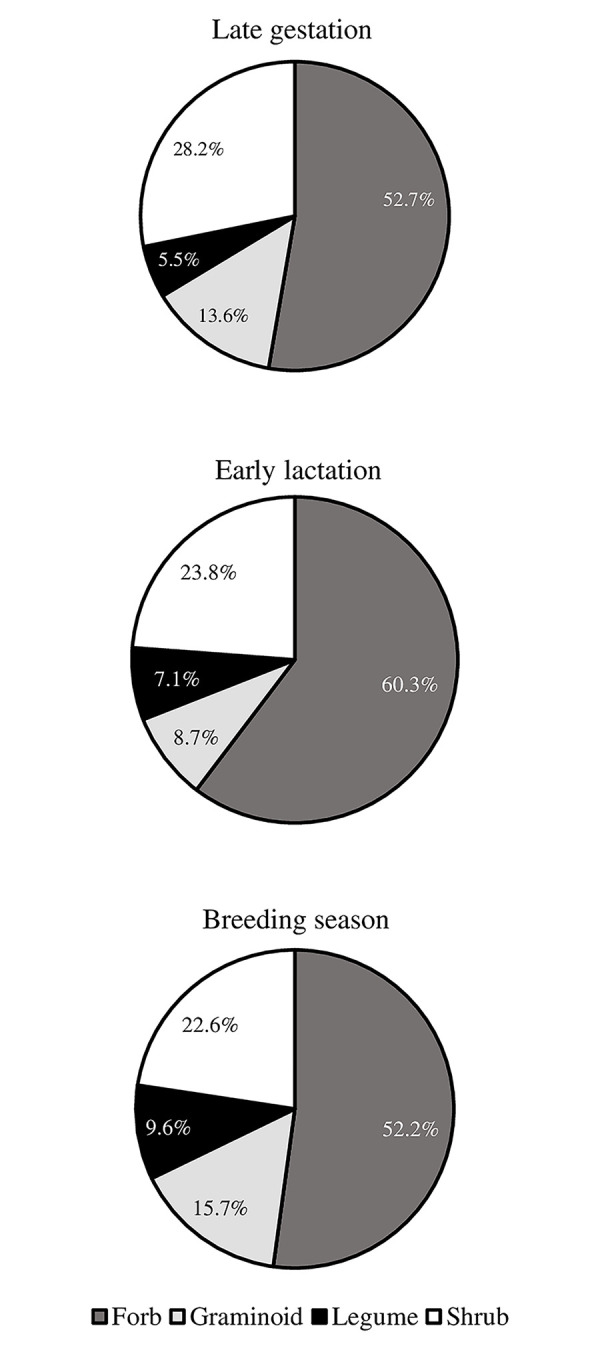
Dietary functional group composition of pronghorn antelope across sampling periods. Average contribution of different plant functional groups (forb, graminoid, legume, shrub) to dietary protein intake for pronghorn antelope from three sampling periods (Late gestation [n = 110], Early lactation [n = 126], Breeding season [n = 115]) across five subpopulations in Idaho, 2018–2019. Legumes only included species from Fabaceae.

**Fig 3:**
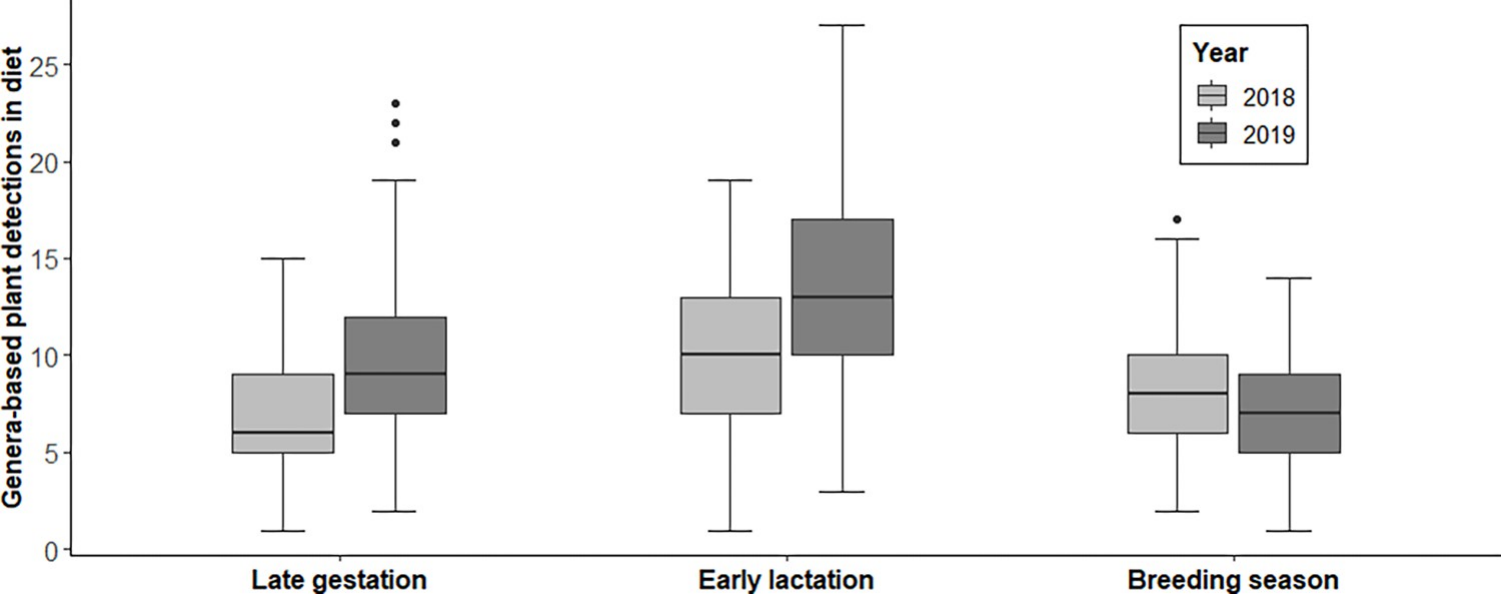
Temporal genera-based plant detections in pronghorn antelope diets. Mean number of genera-based plants detected in pronghorn antelope diets across five study subpopulations by sampling period (Late gestation, Early lactation, Breeding season) in Idaho, 2018–2019.

Dietary functional group composition (i.e., dietary diversity) for pronghorn diets across sampling periods and years was on average 55.1% dietary protein intake from forbs, ranging from 52.2 to 60.3% (Fig 2). For shrubs, mean protein intake was 24.9% (22.6–28.2%), while 12.7% (8.7–15.7%) mean dietary protein intake was derived from graminoids. And finally, diets for the legume functional group composition averaged 7.4% (5.5–9.6%). Pronghorn, across all sites and sampling periods, had a higher dietary diversity in 2019 than 2018 (*SE* = 0.05, *n* = 558, *P* < 0.001; Fig 3). Specifically, pronghorn diets increased in mean genera-based OTUs from 6.87 to 9.42 (*SE* = 0.08, *n* = 558, *P* < 0.001) during late gestation and from 10.10 to 13.40 (*SE* = 0.07, *n* = 558, *P* < 0.001) during early lactation from 2018 to 2019. In addition, late gestation (*SE* = 0.06, *n* = 558, *P* = 0.03) and early lactation (*SE* = 0.05, *n* = 558, *P* < 0.001) sampling, with years combined, were each different in mean OTUs from other periods.

**Fig 4:**
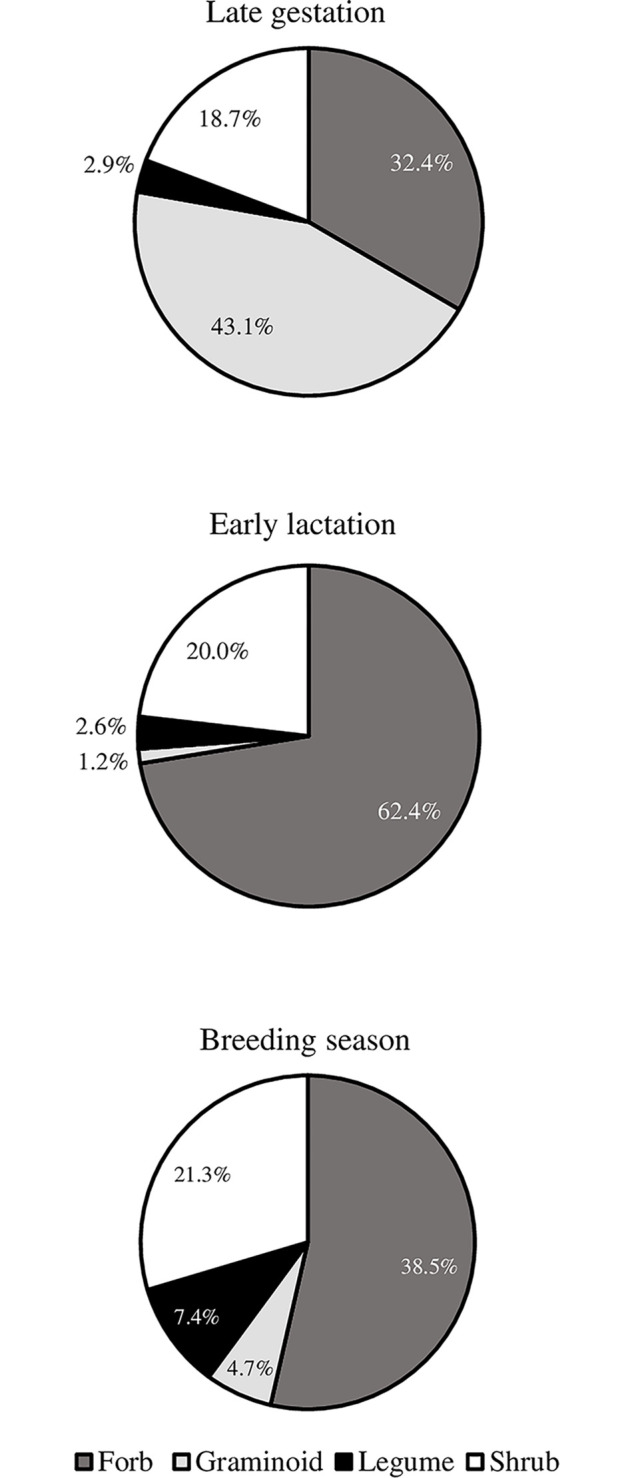
Protein intake by plant functional group across sampling periods for pronghorn antelope. Dietary protein intake by plant functional group (forb, graminoid, legume, shrub) for pronghorn antelope from three sampling periods (Late gestation, Early lactation, Breeding season) across five subpopulations in Idaho, 2018–2019.

Plant DNA barcoding results indicated that pronghorn dietary protein intake was on average 44.4% (32.4–62.4%; Fig 4) from forbs, 20.0% (18.7–21.3%) from shrubs, 16.3% (1.2–43.1%) from graminoids, and 4.3% (2.6–7.4%) from legumes. Moss (*Grimmia* spp.) had one dietary protein intake proportion >1.0% which occurred within the Jarbidge subpopulation during the breeding season in 2019. The highest recorded dietary protein intake was of forbs during early lactation at 62.4% while the lowest was 1.2% from legumes during early lactation. Within subpopulations, dietary protein intake from forbs was generally highest during early lactation, graminoids protein intake highest during late gestation, shrub and legume protein intake highest during breeding season.

## Discussion

We hypothesized that pronghorn would display a greater temporal consumption of forbs beyond peak growing season, would seasonally shift their dietary protein intake to accommodate differing metabolic demands and changes in plant phenology, and would consume differing numbers of plants across sampling periods. Our results demonstrated that dietary functional group composition of pronghorn diets comprised a majority derived from forbs during all sampling periods. We also found that dietary protein intake proportions shifted across sampling periods and that plant genera-based number of detections in the diets varied both within and between years.

We documented protein intake results at two scales, functional group proportions and genus-level proportions within each functional group. Protein intake from forbs constituted the largest dietary proportion during early lactation and breeding season, followed by shrubs, legumes, and graminoids, and second behind graminoids during late gestation. Forbs represent important and nutritious resources for pronghorn and are consumed when available [27]. These plants have been termed production plants where years of high abundance and extended availability and succulence have been correlated to higher fawn recruitment [8]. Shrubs on the other hand, have been labelled as survival plants given that consumption mostly occurs during winter, a period when forage is often low in quantity and quality [8]. Pronghorn in our study demonstrated shrubs are more than just survival plants with seasonal dietary protein intake from this functional group ranging between18.0% and 25.0%.

We found notable shifts in proportions of functional group protein intake between years. The Jarbidge subpopulation decreased their graminoid protein intake during late gestation between years (79.9% to 36.8%), which coincided with an increase in forb protein intake (8.3% to 36.9%). The largest interannual change in legume protein intake occurred during the breeding season within the Birch Creek site from 2.0% to 34.8%. We found forb protein intake within the Little Wood location decreased between years during the breeding season from 60.6% to 18.9%, while shrub protein intake increased from 2.7% to 51.0%, notably from antelope bitterbrush. While dietary breadth of forbs did not change between years for the breeding season, dietary protein intake of prostrate knotweed drastically decreased from 39.3% to 0.6%. This decline may be due to the fact that a portion of the Little Wood pronghorn summer range burned from July through early September of 2018. Fire can have positive effects on herbaceous plant (e.g., forbs) richness and production [28]. Prostrate knotweed does not regenerate vegetatively, limiting its ability to re-colonize, and revegetation timelines post fire vary. It is not known what type of fire regime prostrate knotweed is best adapted to [29] as longer intervals (e.g., 4-year) can negatively impact regeneration [30]. This, in conjunction with fire remediation efforts at the Little Wood site, may have contributed to reduce the availability of prostrate knotweed and thus its presence in the diet.

Plant DNA barcoding yielded 137 plant species detected in pronghorn diets which demonstrated its capacity at identifying species richness, particularly for those plant species present at low proportions in the diet. Finding these dietary components is crucial because they can be important for nutritional balancing and for providing small inputs of nutrients and bioactives that enhance nutrition and health [14, 31] and can significantly influence herbivore fitness [32]. Other studies using microhistological analysis have not detected as many plant species in pronghorn diets [27]. While different methods don’t allow for direct comparisons in this study, it is known that plants that are rare or consumed in “low frequency” are less likely to be discovered and quantified via microhistology [14, 15]. While abundance and biomass cannot be measured with the plant DNA barcoding technique, it is possible to assess species richness within a sample and frequency across samples [33]. Pronghorn increased their dietary diversity from late gestation to early lactation but this parameter decreased during the breeding season. This is likely a consequence of a seasonal response to a varying diversity of plant species available on the landscape, as well as adult female pronghorn increasing their dietary breadth to meet the metabolic demands of lactation. Lactation is the biological period when daily energetic requirements are highest for females across ungulate species [2], where energy requirements increase by 65% to 215% during the first month postpartum [34, 35] compared to nonpregnant females. This increase in metabolic demands was likely met by an increased dietary protein intake of forbs. Forbs are high in crude protein content and are more digestible than other plant functional groups available in the community [36]. In addition, bioactive compounds in forbs and legumes influence animal health and nutrition [37]; phenolic compounds and terpenoids provide antioxidant properties to herbivores that enhance immune function and reduce pathogens and parasitic loads [38, 39], leading to improvements in the efficiency of nutrient use by ruminants [40]. Monitoring protein intake on a temporal scale is central because the availability of crude protein is a key limiting factor in ruminant nutrition and is in declining supply across western U.S. rangelands due to changes in climate [41, 42] and competition with invasive annual grasses (i.e., cheatgrass [*Bromus tectorum*; 43]. Ruminants base their dietary preferences on the association between the orosensorial properties of forages and their post-ingestive consequences [44, 45] and protein-restricted ruminants form strong preferences for foods associated with the supply of protein to the rumen [45].

It is common practice to categorize herbivore diets into the major plant functional groups of forbs, graminoids, and shrubs [8]. We isolated legume as a separate functional group due to the presence of agricultural activities at some of the sites where subpopulations were exposed to. Agricultural crops (i.e., cultivated legumes), notably alfalfa and sainfoin, contributed a lesser proportion of dietary protein intake both spatially and temporally than anticipated given the high nutritional quality of these forages. For instance, pronghorn within the Camas Prairie subpopulation largely resided on private agricultural lands where alfalfa is the primary crop grown. It has been hypothesized in previous work that high summer 2,6-diaminopimelic acid (DAPA; a chemical indicator of forage quality) values were the result of a diet primarily of alfalfa [46], however the highest dietary protein intake of alfalfa was 2.5% in the present study. Thus, the high DAPA values were more likely the result of high dietary protein intake from forbs, which peaked at 77.8% during our study. It appears that in a “sea of alfalfa” available pronghorn foraged on a diversity of forbs instead, likely as a result of rumen and metabolic limitations. The pronghorn rumen microbiome is adapted to a shrub, grass, and forb diet; not to the highly nutritious and high yielding forage legume alfalfa, which is commonly fed to domestic ruminants [47–49]. Ruminants restrict their use of monocultures of alfalfa in part due to the high concentration of ruminal degradable protein in alfalfa, along with a relative insufficient energy concentration, resulting in poor protein utilization by rumen microorganisms [47, 50]. As a result, domestic ruminants limit consumption of alfalfa due to negative post-ingestive effects triggered by bloat and an excess of ammonia in the rumen [51, 52]. Even though pronghorn routinely occupied these alfalfa fields, we believe the perceived direct use (i.e., consumption) may instead likely be indirect or behavioral (i.e., non-consumptive) for a few reasons. Pronghorn fawns depend on a hiding strategy to avoid predators during the first weeks of life [53]. The vertical structure provided by alfalfa may aid in hiding young fawns and positively influence their survival. Pronghorn in the Camas Prairie site may also be capitalizing on irrigated, cooled soils to aid in thermoregulation during summer months. Additionally, there is a lag effect between where and what a pronghorn consumes and their location upon defecation, given the relationship between body size and gut retention times [54].

Sainfoin comprised the majority of legume protein intake with subpopulation proportions at 15.4% and 30.8% within the Camas Prairie and Birch Creek pronghorn, respectively. All other proportions were < 2.0% across sampling periods. We were unable to collect diet data during late gestation in 2018 within the Camas Prairie site, therefore it is not possible to make inferences about interannual changes, but Birch Creek pronghorn demonstrated the largest interannual difference in protein intake of sainfoin from <0.1% to 9.0% and 0% to 30.8% during early lactation and breeding season sampling periods, respectively. This difference could possibly be the result of an interannual crop rotation in the area. Contrary to alfalfa, sainfoin is a non-bloating legume containing bioactive compounds (e.g., condensed tannins) that reduce the incidence of bloat [49] and attenuate the excessive accumulation of ammonia in the rumen through reductions in proteolysis [52]. Collectively, these nutritional benefits and the possible crop rotation could explain the higher protein intake of sainfoin by pronghorn, compared to alfalfa.

Plant DNA barcoding presented limitations in elucidating taxonomic resolution of pronghorn diets. Within each plant functional group “other” categories detected proportions of dietary protein intake that ranged from <0.1% to 76.9% for a few notable families (e.g., Asteraceae, Poaceae, Rosaceae). The “other graminoids” functional group comprised, on average, >80% of total graminoid detections. This family comprised 66 possible species, across 13 genera, with *Bromus* spp. being the sole genus reported. Many of these species were found throughout multiple subpopulation ranges which, in turn, prevented us from inferring differentiation between subpopulations. This lack of genera or species-specific resolution may be a result of technique biases toward detecting non-degraded DNA, which would limit taxonomic identification [14]; heteroplasmy, the presence of multiple copies of genes which can be common in plants [55]; or the reliance on reference libraries, which are continually advancing and becoming more refined as more plant species are barcoded, but are still imperfect [12, 56]. Detections with a genus that contained many species (e.g., >20 possible species) that were present across multiple subpopulation ranges, limited our ability to reduce taxonomic resolution further.

We found diet items that were not native to our study sites and/or potential non-forage items with notable detections being *Cercidiphyllum japonicum* (a tree species native to China and Japan), *Diplostephium ericoides* (a plant endemic to Ecuador), dragon lily (*Dracunculus vulgaris*), and noble rhubarb (*Rheum nobile*). We are unsure how these detections occurred, but most occasions had protein intake proportions far below 1%, with the exception of *Cercidiphyllum japonicum* which was detected four times, three occurring within the Jarbidge subpopulation. Potential non-forage items (e.g., trees) included *Pinus* spp. which were detected in all subpopulations with protein intake as high as 3.0%. It is not known whether or how much pronghorn consume *Pinus* species or if samples were contaminated at collection (e.g., pollen) Contamination, via pollen, or misclassifications may explain the multiple detections of various shrub/tree genera (e.g., *Acer*, *Picea*, *Quercus*) that contributed to the total contribution of shrubs to pronghorn diets in our study areas. We assumed these were errors resulting from contamination or reference library mishaps.

DNA barcoding is particularly useful for detecting the presence of invasive or threatened plants in herbivore diets which can aid in quantifying animals’ impacts as vectors [33, 55]. Graminoid protein was seasonally important, particularly within the Jarbidge site, with protein intake at 58.4% during late gestation. where cheatgrass is present and is of concern in the area [19]. Crested wheatgrass (*Agropyron cristatum*) has been established in areas as a post-fire remediation tool to combat potential cheatgrass expansion and provide forage for domestic cattle. Nevertheless, the proportion of dietary intake of cheatgrass specifically was <1%, at a time when its nutritional quality is highest [57] and demonstrated that pronghorn in the Jarbidge area were likely not noteworthy vectors in spreading the invasive grass.

Finally, plant DNA barcoding has the potential to overestimate the relative contribution of forbs to overall dietary protein, due to their high protein content [12]. In addition, original diet items and specific tissues (e.g., leaves, stems, flowers) contain different densities of DNA and/or are differentially digested, leading to fragmentation of DNA [14]. These issues, in turn, affect interpretations of quantity in the original diet [33, 58]. In response, some have suggested revising interpretation of DNA barcoding results [59] by including control species to generate relative correction factors [60]. As a result, we anticipate plant DNA barcoding will increase in accuracy through in the coming years [61].

## Conclusions

Our results demonstrated that pronghorn dietary functional group composition was a majority of forbs while dietary protein intake shifted seasonally. We examined both measures at the scale of adult female life-stages to find dietary protein intake from forbs highest during early lactation. We believe our results augment previous methods of diet quantification which suggested that consumption of forbs decreased following plant senescence. We also found that while the number of plant species consumed varied by sampling period and year, the plant DNA barcoding method detected a total of 137 species in pronghorn diets, notably many that were consumed in low proportions. We believe this molecular method further confirmed pronghorn’s place as an intermediate feeder, or a group of ruminants that shift feeding behavior according to the availability of forage and season [54], but more closely aligned with a concentrate selector given their dietary protein intake from forbs extended beyond peak growing season. Finally, our separation of legumes as an independent functional group enabled us to document and quantify pronghorn dietary protein intake of agricultural crops, particularly in sites largely dominated by agriculture, where pronghorn occupancy of fields led to perceptions of high agriculture crop consumption.

To our knowledge, this was the first use of a molecular technique to quantify dietary protein intake of pronghorn diets and demonstrated seasonal importance of differing plant functional groups beyond just their presence in diets. We believe plant DNA barcoding presented some limitations, but demonstrated potential elucidating seasonal pronghorn dietary species richness. The barcoding technique is not an estimate of plant abundance, biomass, or volume within a sample; but instead, can measure dietary species richness within a sample and frequency across samples [33] as well as protein intake. Future work directly comparing pronghorn diet results from plant DNA barcoding and microhistological techniques may be beneficial given each method seem to detect diet items that the other misses [12], but was beyond the scope of this study. We believe accuracy and resolution of this molecular technique with improve with continued advancements in reference libraries [61].

## Supporting information

S1 Table Plant DNA barcoding sequencing and relative read abundance results for pronghorn antelope in Idaho. Sequence and read counts of plant chloroplast DNA of pronghorn antelope fecal samples from five subpopulations across three sampling periods (Late gestation [LG], Early lactation [EL], Breeding season [BS]), Idaho, USA. Read abundance was used to generate proportions of dietary protein intake and total percentage of each functional group for each subpopulation, sampling period, and year. Plant names are given as provided by the laboratory.
(XLSX)
